# Traumatic brain injury in older adults: do we need a different approach?

**DOI:** 10.2217/cnc-2018-0001

**Published:** 2018-09-20

**Authors:** Matthew E Peters, Raquel C Gardner

**Affiliations:** 1 Department of Psychiatry & Behavioral Sciences, Johns Hopkins University School of Medicine, Baltimore, MD, 21205, USA; 2 Department of Neurology, University of California San Francisco, CA, 94143, USA; 3 San Francisco Veterans Affairs Medical Center, San Francisco, CA, 94121, USA

**Keywords:** age differences, concussion, geriatric, TBI, traumatic brain injury

Traumatic brain injury (TBI) has been referred to as the ‘silent epidemic’. Luckily, with increasing media attention focused on military- and sports-related TBI, this is no longer the case. However, in the wake of this increased attention and accompanying infusion of federal and private research funding, there has emerged a new silent epidemic: TBI among older adults. Although, progress has been made in decreasing motor vehicle accident related TBI, the opposite trend is seen in older adults with fall-related TBI, who have rising numbers of emergency department visits, hospitalizations and death [1,2]. In fact, as of 2013, adults aged 75 years and older now have the highest incidence of TBI, exceeding incidence in infants [2]. In this editorial, we argue that TBI in older adults is a major public health concern and will require a targeted, age-appropriate approach to clinical care and research. We additionally discuss the challenges of, and potential solutions for, including this population in research.

## Overview of geriatric TBI

Falls, largely from standing height, are the leading mechanism of TBI in older adults, with more women affected, whereas motor vehicle accidents are the leading mechanism of TBI in younger adults, with more men affected [3]. Intracranial changes that occur with aging (e.g., dura adherence to skull, cerebrovascular atherosclerosis and bridging vein fragility), as well as the increasing prevalence of anticoagulant medication use, put older adults at increased risk of intracranial bleeding, even with TBIs that would otherwise be classified as mild [4]. For example, up to 17% of older adults presenting with TBI and a completely normal neurological examination (Glasgow Coma Scale [GCS] of 15) may have evidence of acute intracranial trauma on head computerized tomography (CT) [5]. Pre-existing medical conditions are associated with worse outcomes after TBI and are common in older adults sustaining TBI [6].

On average, older adults with TBI experience higher morbidity and mortality, slower recovery trajectories, and worse functional, cognitive and psychosocial outcomes than younger individuals do [3]. If hospitalized following TBI, older adults are more likely to require extended hospitalizations and to be more severely disabled and functionally dependent after discharge [4,7]. On a more positive note, older adults who are retired prior to TBI report less psychosocial stress post-TBI than younger patients, suggesting that age-related changes in socioeconomic circumstances may be protective [8]. Additionally, a subset of older adults, even those with severe TBI [9–11], may achieve outcomes similar to younger patients [12], indicating that chronological age and TBI severity alone are insufficient to accurately predict outcome.

## Successes & challenges in clinical management

Age has recently been included as a criterion in several TBI prehospital and emergency triage guidelines and several groups are working on TBI treatment guidelines specific to older adults [13–16]. Examples include: geriatric trauma-field triage criteria to optimally identify older adults with TBI who require emergent transfer to a trauma center [13], and neurorehabilitation practices specific to older adults with a focus on removing ‘excess disability’ [14]. In addition, neurocritical care teams increasingly include a geriatrician and the next edition of the *Resources for Optimal Care of the Injured Patient* includes accreditation standards for geriatric trauma care [17].

Despite these successes, many challenges remain. Widely used measures for determining initial TBI severity and estimating prognosis in the acute clinical setting are problematic in older adults. As an example, the GCS is a reliable predictor of morbidity and mortality in younger, but not older, adults who may have an abnormal GCS at baseline or an intact GCS despite accumulating intracranial hemorrhage [3,18,19]. Prognostic models for outcome prediction after TBI (e.g., Corticosteroid Randomization After Significant Head injury CT (CRASH CT), Immediate Post-concussion Assessment and Cognitive Testing [IMPACT]) show poor prognostic performance in older adults with TBI [3]; perhaps because these models do not include key geriatric outcome predictors such as comorbidities, polypharmacy, baseline function and frailty [20]. Recently, the US FDA approved two blood-based biomarkers, GFAP and UCHL1, to aid in the evaluation of mild TBI [21]. However, a small study demonstrated that this GFAP assay is significantly less accurate for identifying CT evidence of intracranial trauma in older versus younger adults with mild TBI [22]. With diagnostic biomarkers rapidly making their way into clinical practice, much more research is needed to guide age-appropriate use.

Lastly, most in-hospital deaths in older adults with TBI occur after elective withdrawal of care [23]. Withdrawal of care decisions are frequently made within 72 h of injury, despite evidence that lack of neurological improvement within 72 h in older adults with severe TBI does not predict long-term recovery among survivors [9]. There are dramatic variations in mortality rates after geriatric TBI between centers [3], suggesting a lack of reliable prognostic indicators to guide acute care decisions in this population. Thus, despite recent and ongoing efforts to improve age-appropriate management of geriatric TBI in the acute setting, there remains much work to be done to implement current evidence into widespread practice and to further develop and refine evidence-based prognostic indicators and care guidelines in order to optimize medical decision making and outcomes in this population.

## The need for a geriatric approach to TBI clinical research

In an effort to study ‘pure TBI’ many prior studies have implemented upper age limits or excluded patients with pre-existing conditions, a practice which preferentially excludes older participants and limits generalizability of findings to this population. Although broad inclusion of older adults in TBI research comes with challenges, we argue that these challenges must, and can, be surmounted if we are to advance care and improve outcomes in the large and rapidly growing population of older adults with TBI. Here, we review some of these challenges and discuss possible solutions.

- TBI diagnosis is challenging in an older adult with head trauma in whom loss or alteration of consciousness may be attributed to TBI, concurrent medical conditions (e.g., stroke, syncope, seizure, dehydration and dementia), or both and therefore may preclude a TBI diagnosis using standard clinical criteria [24,25]. In these ambiguous cases of head trauma, we have chosen to rely on evidence of acute intracranial trauma on neuroimaging studies, such as head CT, for the diagnosis of TBI. Ambiguous cases of head trauma without neuroimaging evidence of intracranial trauma remain a diagnostic challenge and deserve additional dedicated study. Ultimately, a battery of neuroimaging- and blood-based biomarkers may be needed to definitively make the diagnosis in the most challenging or ambiguous cases and more research is needed to identify these optimal diagnostic biomarkers.
- TBI outcome assessment is challenging in an older adult with multiple pre-existing conditions as it may be impossible to isolate the effect of TBI. This is not a new problem in TBI research and has many parallels to the study of TBI in the context of polytrauma. We propose that this challenge can be addressed by systematically measuring and studying the impact of, rather than excluding for, pre-existing conditions and disability. The American College of Surgery Trauma Quality Improvement Program Geriatric Trauma Management Guidelines (all-cause; not TBI-specific) [26] recommends measuring pre-existing comorbidities, functional status and physical frailty in all geriatric trauma patients as these have been proven outcome predictors in geriatric trauma surgery and geriatric medicine inpatient populations [27,28]. Reports of pre-injury disability are already routinely elicited in the context of the most widely used TBI outcomes assessment interview (the Glasgow Outcome Scale Extended [29]) in which patients or their informants are asked to compare current disability to pre-injury disability. Systematically quantifying and recording pre-injury disability, either via patient or informant report, is therefore only a small departure from current practice. Efforts are currently underway to develop improved TBI end points [30]. It is therefore an ideal time to build expert consensus around a set of geriatric-specific TBI end points and common data elements that will facilitate broader inclusion of older adults in TBI research and inform design of clinical trials.
- Frail older adults may be unable to complete outcome assessments. Greater use of proxy informants and study partners, as are commonly used in geriatrics and dementia research, may facilitate inclusion, retention and outcome assessment of the more frail or disabled older patients. Innovative follow-up methods, such as home visits and telemedicine assessments, have the potential to increase follow-up rates and generalizability of results, but will require increased staffing and financial resources. Because older adults with pre-existing conditions may be more likely to have implanted medical devices, MRI clearance protocols (e.g., collection of model and manufacturer information on implanted devices) will be needed to reduce unnecessary exclusions from MRI protocols.

By combining methods commonly used in geriatric research with those already used in TBI studies, the challenges of inclusion of older patients in TBI research can and must be overcome. Only then can the research community achieve generalizability to real-world older adults with TBI, develop better diagnostic and prognostic tools to guide care, design inclusive trials and optimize outcomes.

## Conclusion

This article's title poses the question “Do we need a different approach to TBI in older adults?”. The answer is ‘yes’, but we are not starting from scratch. Clinical endeavors, such as comprehensive, multidisciplinary fall and TBI clinics, are increasing. Geriatric research on conditions relevant to the TBI population (e.g., Alzheimer's disease and related dementias) stand to teach us much about how to design studies focused specifically on older adults with TBI. Older adults with TBI deserve the same advocacy, and focused study, as sports- and military-related TBI. The path forward is increasingly illuminated and the number of us carrying the torch is growing.

## References

[1] (2016). https://www.cdc.gov/traumaticbraininjury/data/index.html.

[2] Taylor CA, Bell JM, Breiding MJ, Xu L (2017). Traumatic brain injury related emergency department visits, hospitalizations, and deaths – United States, 2007 and 2013. *MMWR. Surveill. Summ.*.

[3] Gardner RC, Dams-O'Connor K, Morrissey MR, Manley G (2017). Geriatric traumatic brain injury: epidemiology, outcomes, knowledge gaps, and future directions. *J. Neurotrauma*.

[4] Thompson HJ, McCormick WC, Kagan SH (2006). Traumatic brain injury in older adults: epidemiology, outcomes, and future implications. *J. Am. Geriatr. Soc.*.

[5] Haydel MJ, Preston CA, Mills TJ, Luber S, Blaudeau E, DeBlieux PMC (2000). Indications for computed tomography in patients with minor head injury. *N. Engl. J. Med.*.

[6] Hawley C, Sakr M, Scapinello S, Salvo J, Wrenn P (2017). Traumatic brain injuries in older adults – 6 years of data for one UK trauma centre: retrospective analysis of prospectively collected data. *Emerg. Med. J.*.

[7] Flanagan SR, Hibbard MR, Gordon WA (2005). The impact of age on traumatic brain injury. *Phys. Med. Rehabil. Clin. N. Am.*.

[8] Ritchie L, Wright-St Clair VA, Keogh J, Gray M (2014). Community integration after traumatic brain injury: a systematic review of the clinical implications of measurement and service provision for older adults. *Arch. Phys. Med. Rehabil.*.

[9] Lilley EJ, Williams KJ, Schneider EB (2016). Intensity of treatment, end-of-life care, and mortality for older patients with severe traumatic brain injury. *J. Trauma Acute Care Surg.*.

[10] De Bonis P, Pompucci A, Mangiola A, D'Alessandris QG, Rigante L, Anile C (2010). Decompressive craniectomy for the treatment of traumatic brain injury: does an age limit exist?. *J. Neurosurg.*.

[11] Taussky P, Hidalgo ET, Landolt H, Fandino J (2012). Age and salvageability: analysis of outcome of patients older than 65 years undergoing craniotomy for acute traumatic subdural hematoma. *World Neurosurg.*.

[12] Mak CHK, Wong SKH, Wong GK (2012). Traumatic brain injury in the elderly: is it as bad as we think?. *Curr. Transl. Geriatr. Exp. Gerontol. Rep.*.

[13] Wasserman EB, Shah MN, Jones CMC (2015). Identification of a neurologic scale that optimizes EMS detection of older adult traumatic brain injury patients who require transport to a trauma center. *Prehosp. Emerg. Care*.

[14] Uomoto JM (2008). Older adults and neuropsychological rehabilitation following acquired brain injury. *NeuroRehabilitation*.

[15] Werman HA, Erskine T, Caterino J, Riebe JF, Valasek T (2011). Development of statewide geriatric patients trauma triage criteria. *Prehosp. Disaster Med.*.

[16] Caterino JM, Raubenolt A, Cudnik MT (2011). Modification of Glasgow Coma Scale criteria for injured elders. *Acad. Emerg. Med.*.

[17] American College of Surgeons https://www.facs.org/quality-programs/trauma/vrc.

[18] Peters ME (2016). Traumatic brain injury (TBI) in older adults: aging with a TBI versus incident TBI in the aged. *Int. Psychogeriatr.*.

[19] Salottolo K, Stewart Levy A, Slone DS, Mains CW, Bar-Or D (2014). The effect of age on Glasgow Coma Scale score in patients with traumatic brain injury. *JAMA Surg.*.

[20] Joseph B, Pandit V, Zangbar B (2014). Superiority of frailty over age in predicting outcomes among geriatric trauma patients: a prospective analysis. *JAMA Surg.*.

[21] Food and Drug Administration (2018). FDA authorizes marketing of first blood test to aid in the evaluation of concussion in adults. *FDA News Release*.

[22] Gardner RC, Rubenstein R, Wang KKW (2018). Age-related differences in diagnostic accuracy of plasma GFAP and Tau for identifying acute intracranial trauma on CT: a TRACK-TBI study. *J. Neurotrauma.*.

[23] Turgeon AF, Lauzier F, Simard J-F (2011). Mortality associated with withdrawal of life-sustaining therapy for patients with severe traumatic brain injury: a Canadian multicentre cohort study. *CMAJ*.

[24] Isokuortti H, Iverson GL, Kataja A, Brander A, Öhman J, Luoto TM (2016). Who gets head trauma or recruited in mild traumatic brain injury research?. *J. Neurotrauma*.

[25] Corrigan JD, Cuthbert JP, Whiteneck GG (2012). Representativeness of the traumatic brain injury model systems national database. *J. Head Trauma Rehabil.*.

[26] American College of Surgeons Geriatric Trauma Management Guidelines. *Trauma Quality Improvement Program (TQIP).*.

[27] Peterson MGE, Cornell CN, Paget SA, Allegrante JP (2008). Five-year survival in a cohort of hip fracture patients: the predictive role of pre-fracture health status. *HSS J.*.

[28] Covinsky KE, Palmer RM, Counsell SR, Pine ZM, Walter LC, Chren MM (2000). Functional status before hospitalization in acutely ill older adults: validity and clinical importance of retrospective reports. *J. Am. Geriatr. Soc.*.

[29] McMillan T, Wilson L, Ponsford J, Levin H, Teasdale G, Bond M (2016). The Glasgow Outcome Scale – 40 years of application and refinement. *Nat. Rev. Neurol.*.

[30] Manley GT, Mac Donald CL, Markowitz AJ (2017). The Traumatic Brain Injury Endpoints Development (TED) initiative: progress on a public-private regulatory collaboration to accelerate diagnosis and treatment of traumatic brain injury. *J. Neurotrauma*.

